# Do chiropractic interns use clinical practice guidelines when managing patients with neck pain in France? A feasibility study

**DOI:** 10.1186/s12998-022-00453-1

**Published:** 2022-10-09

**Authors:** Delphine Sorondo, Cyrille Delpierre, Pierre Côté, Nadège Lemeunier

**Affiliations:** 1 CERPOP UMR1295, Université Toulouse III, Inserm, Equipe EQUITY, Toulouse, France; 2 Institut Franco-Européen de Chiropraxie, Toulouse, France; 3 Faculty of Health Sciences, Ontario Tech University, Oshawa, ON Canada; 4 Institute for Disability and Rehabilitation Research at, Ontario Tech University, Oshawa, ON Canada

**Keywords:** Determinants, Guideline’s utilization, Conformity of practice, NAD, Chiropractic interns

## Abstract

**Background:**

In France, we lack knowledge about factors influencing chiropractors’ use of French guideline for managing neck pain and associated disorders (NAD). In particular, we know little about how chiropractic interns use clinical practice guidelines during their training.

**Objectives:**

We aimed to determine the feasibility of conducting a cross-sectional study of chiropractic interns to determine their utilization and conformity with clinical practice guidelines when managing patients with NAD in France.

**Method:**

We developed a web-based questionnaire which included 3 sections: (1) clinical vignettes; (2) determinants of practice and (3) socio-demographic and current practice information. The study was conducted in two phases. The first phase included 2 groups: chiropractors and students (interns). Ten chiropractors reviewed and made recommendations on content (especially clinical vignettes), readability, and flow of the survey. Fifteen interns were invited to pretest the proposed recruitment strategy and determine time needed to survey completion, assess completeness of data collection, and evaluate its readability and flow in students. Due to the low participation of students during the first phase, 20 additional interns were invited to participate and pilot the revised recruitment strategy during the second phase. A group of 20 interns were invited to participate and pilot the revised recruitment strategy during the second phase. Qualitative feedbacks about the recruitment strategy, the content of the questionnaire and the survey process were collected by phone to improve all these steps if necessary.

**Results:**

We collected data from November 2020 to February 2021. In phase 1, 70% of chiropractors (7/10) reviewed the survey and one intern responded (7% participation rate). A revised recruitment strategy was designed and 70% of interns agreed to participate in phase 2. Time to complete the questionnaire was on average 48 m:22 s. Interns evaluated survey content as relevant, comprehensive, covering the range of 4 grades of NAD, and adapted to an intern sample. Five main modifications were recommended by (1) Adjusting survey support; (2) Enhancing communication strategy; (3) Considering interns’ comments about the length of the questionnaire; (4) Modifying 2 determinants not adapted to a French context; (5) Adding a proposal when determinants deal with multidisciplinary management.

**Conclusion:**

Conducting a web-based cross-sectional study of chiropractic interns to assess their utilization and conformity to clinical practice guideline is feasible.

**Supplementary Information:**

The online version contains supplementary material available at 10.1186/s12998-022-00453-1.

Contributions to the literature

- To our knowledge, no study has previously reported on barriers and facilitators that influence the use and conformity with clinical practice guideline by chiropractors for the management of neck pain and associated disorders in France.
- This pilot study describes an effective communication strategy to optimize participation of chiropractic interns in clinical research.
- The emphasis is placed on four main feasibility indicators, namely study process, resources, management, and scientific needs.
- Our study suggests that it is feasible to conduct a web-based cross-sectional study to describe factors influencing and conformity of chiropractic interns with clinical practice guidelines.

## Background

Neck pain and its associated disorders (NAD) are a common cause of chronic pain and disability [1]. In 2015, spinal disorders including neck pain were the fourth leading cause of disability-adjusted life years [2]. From 2005 to 2015, the global prevalence of neck pain increased by 21.1% [1, 3]. Although clinical decisions by healthcare providers and health systems’ efficiency may be improved when informed by evidence-based clinical practice guidelines, implementing guidelines in routine clinical practice remains a challenge [4].

However, a gap exists between care that patient should receive according guidelines and care that patient receive in real practice [5]. This gap was reported among Danish chiropractors where guideline compliance was about 10% for recommendations about acute conditions management and 43% for chronic conditions [6].

In France, the first evidence-based chiropractic guidelines were published in 2017 and focused on the assessment and management of neck pain and associated disorders (NAD) [7–13] and Information about its use of the guidelines and determinants of its utilization are necessary to ensure and improve quality of patient care. To date no assessment of guidelines utilization by chiropractors in France has been conducted.

As reported in our recent coping review of the literature, any determinants influence the use of clinical practice guidelines by healthcare providers [14]. The review identified few patients’ determinants, highlighting that literature on guideline use primarily focuses on clinicians. Moreover, we found no differences in determinants across a range of healthcare professionals. Overall, the identified barriers and facilitators illustrated divergent views about guideline use. For example, users of guidelines perceived recommendations as adaptable to daily practice because they are relevant, useful, accessible, concise, and clear. Conversely, non-users considered that guidelines did not improve the quality of health care because they were restrictive, cumbersome, theoretical, too numerous and time consuming.

A possible solution to improve the routine use of evidence-based practice is to implement guidelines in training curriculums during clinical training of future healthcare professionals. This needs evidence on current guidelines utilization, determinants of its utilization, and compliance with its recommendations to ensure and improve the quality of patient care.

However, conducting such studies requires careful planning to develop a sound framework to optimize the recruitment strategy, to upgrade the content of the questionnaire and to improve the survey process. Its feasibility first needs to be established to maximize internal validity. The aim of current study was to investigate the feasibility of conducting a cross-sectional study of guideline utilization by chiropractic interns (undergraduate final year students taking care of patients while being supervised by clinicians). Our study aimed to assess (1) implementation barriers, and (2) study processes, and resource needs by measuring the following feasibility metrics: (a) recruitment strategy; (b) participation rate; (c) time to complete the questionnaire; (d) completeness of the data collection.

## Method

### Context

The Institut Franco-Européen de Chiropraxie (IFEC) is the only chiropractic college in France graduating chiropractors and located at Ivry-Sur-Seine (Paris) and Toulouse (South of France). In France, chiropractic studies are available after bachelor’s degree and lasts 5th years. During the second semester of the 4th year and all the 5th year, students are interns at IFEC clinics, where they co-manage patients with experienced clinicians. In France, chiropractic guidelines for the management of NAD are taught throughout the student's academic career. Each undergraduate course delivered since 2017 on the management or diagnosis of NADs refers to those recommendations [12].

### Study design

We conducted a feasibility cross sectional study and followed the Strengthening The Reporting of Observational Studies In Epidemiology (STROBE) statement to report the study [15, 16] (Additional file 1). Our feasibility study was initially designed to include one phase. However, due to the low participation rate, a second phase was conducted to improve, implement, and test a new recruitment strategy and a different survey support identified as no efficient during the first phase. We piloted the first phase of survey instrument in November 2020, in two samples. The first sample included 10 practicing chiropractors. The second sample included 15 interns (in the 5th and final year of training). The initial survey was designed in Google Forms [17]. Then, survey support switched from Google Forms [17] to SurveyMonkey [18]. This version was implemented in the second phase between February 12th to 19th 2021 to determine the feasibility of conducting the study online with Survey Monkey support [18] on a third sample formed by 20 chiropractic interns.

### Feasibility indicators

Three feasibility indicators were assessed in this study: processes, resources and management [16]. Processes include methodological aspect as recruitment of participants and participation rate, attrition, and relevance of inclusion criteria. Resources evaluated time and problems measured by data completion rate and utilization of platform and software. Management measures data variability and adverse events to estimate data and potential participant issue.

### Study sample, recruitment, and data collection

#### Practitioners’ panel

To evaluate the content of the survey, a convenient sample of ten chiropractors aware of the NAD guidelines were invited to join the practitioners’ panel. There were selected by the first author based on their age, and diversity and locations of their private practice (urban or rural). All chiropractors included in the panel were chiropractic interns’ supervisors at the clinic and employed by IFEC. Potential participants were invited by email sent by the first author on November 12th, 2020. The online survey was available for 3 weeks from November 12th, 2020 to December 3rd, 2020. Three reminders were sent on November 19th and 26th, 2020 and on December 1st, 2020. If a positive response was sent by email, a weblink to the survey was proposed for potential participants in a second email. This email also contained discussion about materials and resources presented as a preliminary step for a future study. Consenting participants had 45 min to review a questionnaire designed for chiropractic interns. They were asked to espouse the viewpoint of an intern.

Only the first version of the survey was proposed by an online Google Forms support. Participant could only participate once. They were free to give feedback by email or phone call to the first author after completion.

#### Chiropractic interns’ group

A first sample of 15 chiropractic interns were randomly selected using the annual list of enrolled students provided by the institution’s administration by electronic drawing in Excel (Microsoft Corporation (2018)). An email introducing the study was sent by the first author to invite selected interns to participate in the study. Interested participants then received a Google Forms weblink to the survey. Participants were asked to read an information letter, provide consent, and complete the survey. The survey was available for 3 weeks from November 16th, 2020, to December 7th, 2020. Three reminders were sent on November 23rd and 30th and on December 4th, 2020. Students could participate only once, and their feedbacks was collected by emails or phone calls.

Our first recruitment strategy yielded a low participation rate (n = 1/15). Then, a second phase was designed to improve the recruitment strategy that included a new communication strategy and tested in a second sample of 20 randomly selected chiropractic interns (n = 10 in both locations). Interns who were included in the first sample were not eligible from the second sample. The second sample was invited to participate by email using IFEC email addresses. The link was delivered to interns by email invitation with an informative 4-min-long video featuring potential participants about the study objectives and methodology. A study announcement was posted on professional Facebook groups of each academic year, 1 week before the email invitation. Interns were instructed to complete it all at once using the weblink. Another modification was implemented by changing the questionnaire electronic platform from Google forms to Survey Monkey [18]. Participants completed the questionnaire online. The survey was available for 1 week from February 12th, 2020, to February 19th, 2020. Leaving the online questionnaire and returning to it later was not possible. The invitation email was sent on February 12th, 2020. Two reminders were sent, one on February 16th, 2021, and another on February 19th, 2021. Time required to complete the questionnaire was estimated about 45 min by the software without any follow up. Potential participants were aware that they had to participate to a phone-call with first author to report their feedbacks after having completed the survey.

Video link available on www.youtube.com/watch?v=Y__BJyBwsPE

From February 19th, 2021, to February 24th, 2021, a 10-min phone call was scheduled by the first author with each participant to evaluate the feasibility of administering the survey and to debrief about their experiences and concerns about the usability of the survey. Participants were invited to provide feedback about the questionnaire by answering five questions in a semi-structured interview: (1) perception of length of completion time; (2) comprehension of questions; (3) understanding of vignettes; (4) presence of an ambiguous question; (5) possible additions to the questionnaire.”

#### Survey questionnaire

The first section of the questionnaire included the informed consent form (Additional file 2*written in French*). The opening page described the: (1) study objectives; (2) participants tasks; (3) risks, inconveniences, discomforts, and constraints; (4) benefits for the participant; (5) confidentiality of data; (6) voluntary contribution of participant; (7) diffusion of data; (8) thank you and compensation; (9) responsible for research project; (10) retraction form if needed.

If a potential participant refused to participate indicating her/his non-consent, she/he was redirected to a web page containing four quick questions about sex, age, step of her/his progress in clinical training and use of guidelines (yes/no).

The survey included three additional sections: (1) clinical vignettes assessing the conformity of practice, (2) seven sections with 85 questions about barriers and facilitators of guidelines’ utilization, and (3) nine questions on socio-demographic data.

The order of the three parts survey was determined by a 5-member working group composed of researchers and chiropractors (AL, FBC, CHG, GB, MP). They agreed to organize the questionnaire from the part requiring the most attention to the part that required the least. For the final version, survey was organized as: Part 1) clinical vignettes; Part 2) questionnaire about utilization influencers; and Part 3) Sociodemographic questions.

#### Clinical vignettes assessing the conformity of practice

##### Development of vignettes

Four clinical vignettes describing patients with different clinical presentation and severity of neck pain were developed according to The Bone and Joint Decade 2000–2010 Task Force classification [19] (Additional file 2*written in French – “cas A” corresponding to grade 2, “cas B” corresponding to grade 1, “cas B suite” corresponding to grade 3 and “cas D” corresponding to grade 4*). The vignettes were developed by the first author (DS) and two expert researchers and chiropractors (PC and CC). Questions used to measure interns conformity were developed according to the algorithm proposed in the French guidelines [12, 20, 21]. Answer options respected the algorithm of the French guideline concerning the management and assessment of NAD by chiropractors [11] (Fiche memo and algorithms available on https://www.ifec.net/recommandations/).

Draft vignettes were reviewed by five researchers and chiropractors (AL, FBC, CHG, GB, MP) who were familiar with the guidelines. Modifications to the four vignettes were made to improve the clarity of the text, add missing information, and remove potential ambiguities. Discussions were conducted to verify that content of guidelines were fully covered by the clinical cases.

#### Questionnaire about facilitators and barriers of guideline utilization

##### Survey design

Questions about barriers and facilitators of guideline utilization were based on our scoping review [14] (Additional file 2 written in French). The Theory of planned behavior [22] was the most commonly used framework to report determinants following 4 categories: (1) Behavioral Intention, (2) Attitudes toward behavior, (3) Subjective Norm and (4) Perceived behavioral control [23]. Each determinant identified by the scoping according to the Theory of Planned Behavior [22] was translated in French and rephrased as a question, and the questionnaire was organized in 7 parts reporting: (1) Practitioner perception of his own practice (17 questions); (2) Practitioner viewpoint about multidisciplinary relationship (7 questions); (3) Patients’ types with whom practitioners use guidelines ( 8 questions); (4) financial incentives (3 questions); (5) perceived usefulness of guideline by practitioners (21 questions); (6) Practitioner viewpoint about guideline content (21 questions); (7) identification of practitioners’ lack of knowledge (6 questions).

### Survey reviewing

All questions were reviewed by the working group who identified unclear questions and questions that were not relevant to chiropractic practice in France. The initial questionnaire was submitted to 10 practicing chiropractors for review. The final questionnaire included 83 questions on perceived barriers and facilitators with answers reported in using a Likert scale with 4 proposals: “agree”, “rather agree”, “rather disagree”, “disagree”.

#### Socio-demographic data and guidelines use indicators

Sociodemographic data were collected by 9 questions (Additional file 2*written in French*) including gender, age, academic year, and number of patients treated at this stage in the clinic. Indicators of guidelines utilization in clinical practice was measured using accepted international recommendations including French ones [7–13]. According to the question about which guideline they used, participants were free to complete an open-ended question, if the guideline was not included in the proposed list. The use of guidelines was also inquired by asking participants if they used it to: (1) make a diagnosis; (2) choose a treatment; and (3) evaluate prognosis. Finally, the last question asked whether they attended a course about guidelines utilization during their undergraduate program.

Time required to complete the questionnaire was estimated at 45 min by the Survey Monkey algorithm.

#### Statistical analysis

Descriptive data of practitioners’ panel et undergraduate chiropractors were estimated respectively in Tables 1 and 2. The three feasibility indicators were described. Process indicators were estimated by measuring participation rate and providing reasons of non-participation or study retraction. Resource indicators were measured by data completion and completion time. Management indicators were estimated, helped by participants feedback about potential adverse events in a qualitative way.

**Table 1:** Descriptive data of practitioners’ panel

	Practicing chiropractors (n = 7)
Gender, male % (n)	71.4% (n = 5)
Age, mean (± SD)	41.6 (15,3)
**Experience—years of practice, % (n)**	
Less than 5 years	28.6% (n = 2)
Between 5 and 30 years	57.1% (n = 4)
More than 30 years	14.3% (n = 1)
France practice since 2017, % (n)	100% (n = 7)
**Practice location, % (n)**	
Urban	57.1% (n = 4)
Sub-urban	42.9% (n = 3)
**Type of practice, % (n)**	
Alone	57.1% (n = 4)
With other chiropractors	28.6% (n = 2)
With other healthcare practitioners	14.3% (n = 1)
IFEC employee since 2017, % (n)	71.4% (n = 1)
AFC members, % (n)	85.7% (n = 6)

*IFEC* Institut Franco-Européen de Chiropraxie (french chiropractic college), *AFC* Association Française de Chiropraxie (french chiropractic assocication)

**Table 2:** Descriptive data of chiropractic interns

	Chiropractic interns (n=13)
Gender, % (n)	46.15 % male (n=6)
Age (mean/SD)	24 (0.9)
**Number of cares delivered, % (n)**	
0 > 100	23.08% (n=3)
101 > 200	53.85% (n=7)
201 > 300	23.08% (n=3)
301 > 360	0

## Results

### Processes feasibility indicators

**Fig. 1:**
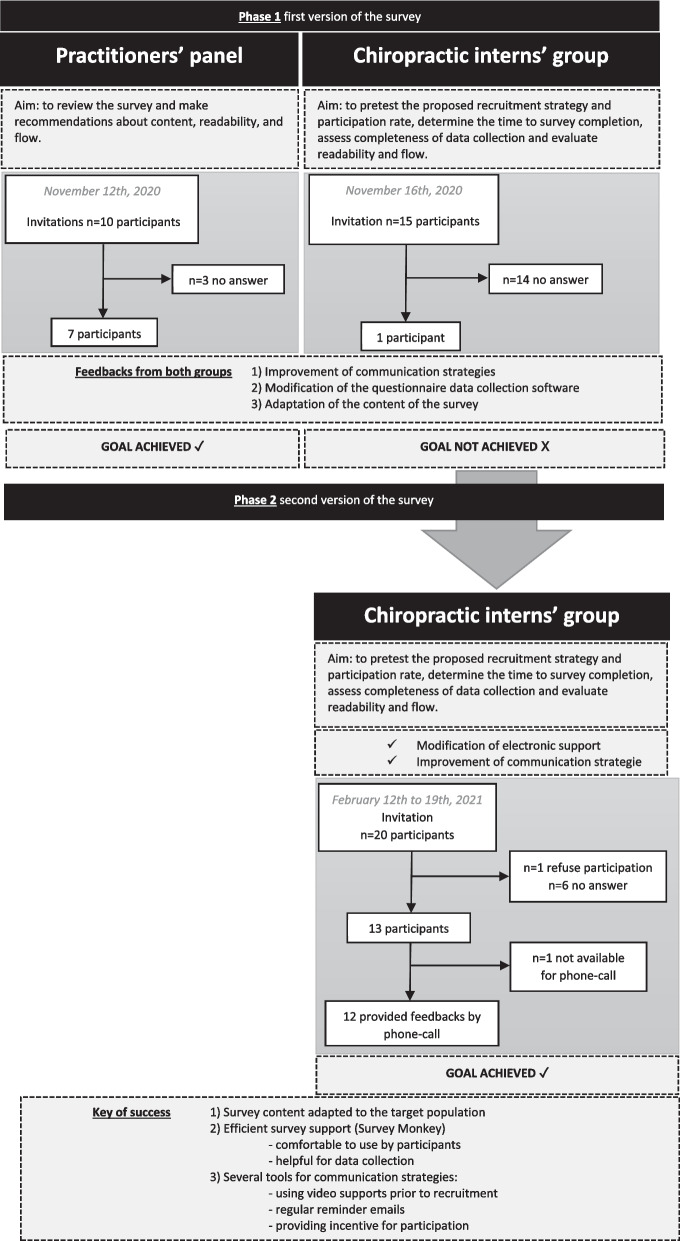
Flow chart for partitioners’ panel and chiropractic interns’ group

Overall, forty-five potential participants including 10 chiropractors and 35 undergraduate chiropractors were invited to participate to the study (Fig. 1).

#### First phase

In the first phase, the participation rate of the practitioners’ panel was 70% (n = 7/10) and all completed the questionnaire. Five participants reported that Google Forms was not reliable or not easy to use.

Regarding chiropractic interns’ group, participation rate for the first phase was 7% with only 1 participant (n = 1/15). The email-only recruitment strategy was not effective. The following difficulties in completing the questionnaire were reported with Google Forms: (1) support not enough intuitive, (2) loop while answering the questionnaire, (3) difficulties to quit and return without losing responses. For these reasons, this platform was dropped.

#### Second phase

Participation rate for the second phase was 65% (n = 13/20). Survey Monkey support offered to participants an easier-to-use interface. Seven individuals refused to participate and only one provided reasons for non-participation (did not feel comfortable with French recommendation about the management of NAD patients and therefore preferred not to participate to the study). All participant completed the questionnaire. There was no missing data.

All the student participants (n = 13/13) were already familiar with this material as it had been used several times during their training. During completion no failures were reported. This interface was suitable for a larger study.

### Resources feasibility indicators

#### First phase

Following the recommendations of the practitioners panel (n = 7), determinants about hospital-based practice were removed from the survey because they are not relevant to French context. However, an item about managing a high volume of NAD patients was added. In the final version of the questionnaire (Additional file 2*written in* F*rench*), determinants about income of practitioners were reworded to fit a French context because it is kept confidential in France usually and not communicated to others. The final version of the questionnaire included 7 Sects. (83 questions) deemed relevant to chiropractic practice in France. This version was implemented in a second phase completed by undergraduate chiropractors. Understanding of vignettes was judged as fitting with recommendation and sufficiently detailed to complete the survey by both clinicians and student. Comprehension of questions was reported as acceptable for both.

#### Second phase

On average, participants spent 48 m:22 s to complete the questionnaire. The completion of the survey took too long time to 50% of interns (n = 6/13).

Most participants (n = 12/13) provided feedback by scheduled phone-call. One participant was finally not available.

Chiropractic interns (n = 13/13) recommended to add a response option to the Likert scale. After discussion and consensus, the item “not enough experienced to answer the question” was added. They also reported difficulties with questions about multidisciplinary approach because some of the students were not experienced with managing patients in a multidisciplinary environment. Comprehension of other questions was validated by the group answering the phone-call. They also confirmed they had enough information reported by clinical vignettes to complete the survey. Frustration feelings was emerging from the use of a Likert scale with 4 options by 4 chiropractic interns.

### Management feasibility indicators

Chiropractic interns represent the target study sample. The initial communication strategy was based on inviting potential participants by email. On the email, a weblink was included to have access to information about the study leading to the consent forms and finally, if agreement, the survey. For the first phase of invitation, only 1 participation over 15 invitations was registered.

In phase 2, communication strategies were redesigned in collaboration with IFEC communication department. Supports were diversified and dissemination optimized. The descriptive 4-min-long video done by the first author was watched by all the participants (n = 13/13). They had read the post on Facebook. All the participants admitted that reward presented in the video encouraged them to contribute to the study. According to their future practice, winning a massage machine was deemed a non-coercive incentive to participate.

Concerning the planification of study participation, because of the length of the questionnaire, advice was given by undergraduate chiropractors to implement the survey during courses linked with guidelines learning. In this way, time to perform the task would be acceptable and interest for the survey could improve.

No adverse events were reported.

## Discussion

Our feasibility study demonstrates that the conduct of a cross-sectional study is feasible in chiropractic interns enrolled at IFEC. Our methodology is appropriate to measure conformity of practice and the association between compliant practice and chiropractic interns’ characteristics concerning the management and the assessment of NAD patients. Results from the second phase of invitation suggest that recruitment strategy and data collection method could be implemented in a larger study.

Two modifications were made to the content of the questionnaire: (1) modifying the question about remuneration France, and (2) deleting the question about hospital-based practice bjectiven France.

The participation rate in the feasibility study was acceptable using the revised recruitment strategy. Regarding participation rate of chiropractic interns’ group, email invitations alone were not sufficient. Students received a number of emails each day, we assume that they did not pay attention to an additional email about something they did not hear anything. A revised recruitment strategy that included short videos in a question-and-answer format help to keep students' attention, and a lottery to win a massage device improve participation. Furthermore, disseminating videos on emails and on student social network groups may have improved participation. Regarding the online questionnaire it seemed that an access for 1 month was sufficient. Regarding reminders, sending 4 email reminders at the beginning of each 4 weeks of the study seemed appropriate.

The time needed to complete the questionnaire provides an estimate to plan for the cross-sectional study. About 50 min to complete the survey is feasible during a lecture. However, the same duration would likely not be acceptable without incentives for a chiropractor in private practice [24, 25]. Other options could be implementing the survey in professional education or at graduation ceremony.

Electronic support has been adapted depending on feedbacks obtained from practitioners’ panel and chiropractic interns’ group. Survey Monkey was more intuitive to elaborate and to answer the survey than Google Forms support.

Clinical vignettes were validated by an expert panel of 10 practicing practitioners as relevant, sufficiently comprehensive, assessing the 4 grades of NAD. Feedbacks from chiropractic interns were homogeneous, they did not express a desire to change the vignettes. They were judged by each group as enough detailed to represent a real clinical situation of NAD. Questions associated with this part was understandable, and consistent with clinical cases. According to questions regarding determinants about guideline utilization, after removing French context non-adapted questions of the first version, chiropractic interns with the smallest experience have faced problems with questions about multidisciplinary patient management. Adding a proposal as “not enough experienced to answer the question” was the solution to continue the question and to express a new determinant as being not enough experienced to use recommendations. The last part about sociodemographic questions did not receive any comment, no questions were considered embarrassing by chiropractic interns. No revisions were requested by the expert panel or by the chiropractic interns’ group. In the main study, this part will be conserved in this state.

### Limitations and strengths of this study

This cross-sectional study has some limitations. According to the instrument used in the questionnaire, the inability to provide a neutral response led to students’ frustration. These negative feelings could have impacted retention and increase the rate of incomplete questionnaires. Moreover, only one student justified why he did not participate to the study. He thought that he had not the level to answer questions about NAD French guideline. Not enough information could explain nonparticipation and so evaluation of selection bias is not possible. The duration to complete the survey could be an important limitation to chiropractors in a private practice participation because most of them spent 30 min for a patient visit. Probably the professionals are not ready to compromise so much of their practice time. In the future, this limitation must be considered to encounter a sufficient participation rate. A proposed solution would be to implement the survey in the annual general meeting of the professional association.

This feasibility study has also strengths. First, the content of the questionnaire the was informed by our scoping review of the literature [14]. Although, a theoretical underpinning and potentially missing questions to uncover pertinent barriers/facilitators to guideline use could be two major weaknesses of this work. Second, the protocol has been established with the aim of minimizing the constraints imposed on the participants. Third, the survey was enthusiastically received by participants who devoted themselves seriously to its improvement. Finally, this feasibility study has been elaborated in two phases in order to implement internal validity. The two major modifications were: (1) Redefining the original communication strategy to a more effective recruitment; and (2) Adapting questionnaire support to avoid completion dysfunctions.

### Future research

During the first half of 2021, the protocol was implemented in a larger sample of approximately 200 undergraduate chiropractors to study guidelines utilization influencers and assess conformity of undergraduate chiropractors’ practice when managing NAD patients. The study is now in progress.

## Conclusion

This feasibility study has provided valuable information helping improve materials that will be used in the main study. This protocol is feasible and well received by chiropractic interns which are targeted population for a future cross -sectional study. Tools have been modified helped by chiropractors and interns’ feedbacks. The last version is adapted and ready to be implemented in a larger group.

## Supplementary Information

**Additional file 1**. STROBE Statement—Checklist of items that should be included in reports of cross-sectional studies.

**Additional file 2**. Enquête nationale stagiaires en centres de soins (PILOTE) - Diagnostic & prise en charge des cervicalgies en chiropraxie.

## Data Availability

The aggregated data are accessible to researchers upon reasonable request for data sharing to the corresponding author.
