# Supplementary material for: Do chiropractic interns use clinical practice guidelines when managing patients with neck pain in France? A feasibility study
Source: Chiropr Man Therap. 2022 Oct 9;30:43. doi: 10.1186/s12998-022-00453-1 (PMC9548113; doi:10.1186/s12998-022-00453-1)
Supplement: Supplementary file 2 — Additional file 2. Enquête nationale stagiaires en centres de soins (PILOTE) - Diagnostic & prise en charge des cervicalgies en chiropraxie. [file 12998_2022_453_MOESM2_ESM.pdf]

Enquête nationale stagiaires en centres de soins (PILOTE) - Diagnostic & prise en charge des cervicalgies en chiropraxie

INFORMATIONS ET CONSENTEMENT

Ce projet de recherche vise à observer votre pratique clinique quotidienne et à étudier les caractéristiques et déterminants qui pourraient influencer vos décisions cliniques dans la prise en charge des cervicalgies. Votre participation à ce projet serait grandement appréciée pour faire avancer les connaissances sur les pratiques chiropratiques en France.
Le but de cette notice d'information est de vous permettre de comprendre ce qu'implique votre éventuelle participation à ce projet de recherche. Prenez donc le temps de la lire attentivement et n'hésitez pas à poser toutes questions que vous jugerez utiles au chercheur principal, Delphine Sorondo, via cette adresse mail dsorondo@ifec.net

Objectifs (but du projet)
L'objectif principal de ce projet est d'observer et de décrire la pratique clinique des chiropracteurs français ainsi que celle des étudiants en stage clinique et celle des encadrants pédagogiques au centre de soins de l'IFEC concernant le diagnostic et la prise en charge des patients atteints de cervicalgies.
Cette observation va se faire à l'aide de vignettes cliniques informatisées qui décriront au chiropracteur 3 cas cliniques différents illustrant des situations concrètes pouvant être rencontrées en pratique libérale. Cette étude inclut également un questionnaire qui permettra de faire l'état des lieux des caractéristiques ou déterminants personnels pouvant influencer la pratique clinique du chiropracteur.
Tâche (Ce que l'on attend de vous)
Votre participation à ce projet consiste à répondre au questionnaire et aux 3 cas cliniques dont le remplissage prend environ 40 minutes. Vos réponses doivent être spontanées reflétant ainsi le plus fidèlement possible votre pratique quotidienne. Il n'y a pas de bonne ou de mauvaise réponse. Imaginez-vous immergé dans votre pratique clinique quotidienne pour répondre aux questions. Le projet ne comporte qu'une unique participation de votre part qui s'effectuera via un ordinateur. Nous vous conseillons de vous installer dans un endroit calme où vous pourrez rester concentré du début à la fin de l'enquête que vous réaliserez en une seule fois.
Risques, inconvénients, inconforts, contraintes
Aucun risque n'est associé à votre participation. Le temps consacré au projet, soit environ 40 minutes pour le remplissage du questionnaires et documents annexes, demeure la seule contrainte.
Bénéfices
Le fait de participer à cette enquête vous donne l'opportunité de vous exprimer anonymement sur votre pratique quotidienne en cabinet. Cette évaluation via vignettes cliniques et questionnaire vous permettra de prendre conscience de l'influence que peuvent avoir certains facteurs sur votre pratique actuelle. Votre participation contribuera aussi à l'avancement des connaissances sur la profession à grande échelle en France dans le but d'adapter les futures communications professionnelles et outils afin qu'ils soient le plus proche possible de votre pratique quotidienne et de vos attentes.
Confidentialité
Les données recueillies dans le cadre de ce projet sont entièrement confidentielles et ne pourront en aucun cas mener à votre identification. Votre confidentialité sera assurée grâce à un code numérique qui sera attribué à chaque participant. Lors du traitement et de l'analyse des données, seuls ces codes seront utilisés. Les résultats de la recherche, qui pourront être diffusés sous forme d'articles scientifiques, ne permettront pas d'identifier les participants.
Les données recueillies seront conservées sur l'ordinateur du chercheur principal et seront protégées par un code d'accès (mot de passe). Les seules personnes qui y auront accès seront le chercheur principal et les co-chercheurs. Toutes ces personnes ont signé un engagement à la confidentialité. Les données seront détruites 5 ans après l'étude et ne seront pas utilisées à d'autres fins que celles décrites dans le présent document.

Participation volontaire
Votre participation à cette enquête se fait sur la base du volontariat. Vous êtes entièrement libre de participer ou non, de refuser de répondre à certaines questions ou de vous retirer à tout moment, sans préjudice et sans avoir à fournir d'explication. La procédure de rétraction est décrite en début d'étude dans le consentement et la personne à contacter est Delphine Sorondo, le chercheur principal (dsorondo@ifec.net). Dans ce cas, les données recueillies seront immédiatement détruites. Le chercheur se réserve aussi la possibilité de retirer un participant en lui fournissant des explications sur cette décision.
Diffusion
Les résultats de cette recherche seront communiqués au cours du premier trimestre 2021. Elle fera l'objet de la rédaction d'un article scientifique et pourra être présentée lors de colloques ou conférences scientifiques internationales. Enfin, cette étude s'inscrit dans un projet de thèse en épidémiologie.
Remerciements et récompense
Votre collaboration est précieuse. Nous l'apprécions et vous en remercions grandement.
Tous les participants à l'étude pourront, s'ils le souhaitent, donner leur adresse mail à la fin du questionnaire pour être inclus à un tirage au sort. Cette adresse mail ne sera pas reliées aux réponses données, elle sera uniquement prise en compte pour le tirage au sort. Le gagnant de ce tirage sera informé par mail, et aura accès à une liste de séminaires de formation continue en chiropraxie ayant lieu en France. Il pourra choisir dans cette liste celui auquel il voudra participer. Les frais de participation au séminaire hors frais de déplacement et vie sur place seront remboursés.
Responsable de la recherche
Pour obtenir de plus amples renseignements ou pour toutes questions concernant ce projet de recherche, vous pouvez communiquer avec Delphine Sorondo. (dsorondo@ifec.net)
Cette recherche est approuvée par le comité d'éthique de l'Institut Franco-Européen de Chiropraxie et un certificat portant le numéro CE 2020-08-28-1 a été émis le 28/08/2020
Pour toute question ou plainte d'ordre éthique concernant cette recherche, vous pouvez contacter le comité éthique d'établissement de l'Institut Franco-Européen de Chiropraxie par mail : comite.ethique@ifec.net

CONSENTEMENT ÉCLAIRÉ
Nous, Delphine Sorondo (chercheur principal) et Nadège Lemeunier, Pierre Côté, Cyrille Delpierre (co-chercheurs), nous engageons à procéder à cette étude conformément à toutes les normes éthiques qui s'appliquent aux projets comportant la participation de sujets humains.

Consentement du participant
Je confirme avoir lu et compris la lettre d'information au sujet du projet « Analyse de la pratique des chiropracteurs français : quelles caractéristiques ou déterminants personnels définissent votre pratique quotidienne dans le diagnostic et la prise en charge des cervicalgies ? ». J'ai bien saisi les conditions, les risques et les bienfaits éventuels de ma participation. Réponses ont été apportées à toutes mes questions. J'ai disposé de suffisamment de temps pour réfléchir à ma décision de participer ou non à cette recherche. Je comprends que ma participation est entièrement volontaire et que je peux décider de me retirer en tout temps, sans aucun préjudice.

Formulaire de rétractation
A tout moment vous pouvez interrompre votre participation à cette étude sans raison précise en envoyant un mail à Delphine Sorondo (dsorondo@ifec.net) l'informant de votre rétraction. Vos données seront alors détruites et non incluses dans les résultats.

* **J'accepte donc librement de participer à ce projet de recherche :**

- ◯ OUI
- ◯ NON

Merci d'avoir répondu à l'appel

Afin de compléter nos statistiques pouvez vous répondre à ces 3 questions s'il vous plait ?

Quel âge avez-vous ?

- 17 ans ou moins
- 18 à 20 ans
- 21 à 29 ans
- 30 à 39 ans
- 40 à 49 ans
- 50 à 59 ans
- 60 ans ou plus

* Êtes-vous... ?

- Une femme
- Un homme
- Autre (précisez)

* Vous exercez votre proffession :

- Seul(e)
- En collaboration

Enquête nationale stagiaires en centres de soins (PILOTE) - Diagnostic & prise en charge des cervicalgies en chiropraxie

VIGNETTES CLINIQUES - CAS CLINIQUE A

Dans cette partie, nous allons vous proposez des situations cliniques sous forme de cas clinique concernant le diagnostic et la prise en charge des douleurs cervicales en chiropraxie.

Anamnèse :
Patiente de 25 ans qui se présente avec des douleurs cervicales apparues spontanément au réveil il y a 5 jours. Elle cote sa douleur à 6/10. Aucun traumatisme ne peut expliquer sa douleur. Elle a déjà ressenti ce type de douleur il y a 5 ans. Elle est localisée au niveau de l’arrière du cou mais aussi à l’avant et se diffuse dans l’épaule droite jusqu’au moignon et dans la moitié droite du dos jusqu’au milieu des omoplates. Elle a du mal à s’endormir, ne trouvant pas de position antalgique mais une fois qu’elle trouve une position de confort (tête bien calée), elle s’endort et ne se réveille pas. Les anti-inflammatoires non stéroïdiens la soulagent en diminuant sa douleur à 4/10. Son médecin généraliste l'a mise en arrêt maladie pour une semaine à la suite de ses douleurs. Sa douleur l’angoisse car elle ne peut pas travailler, l’empêche de conduire et de faire des activités en général par exemple faire sa gym matinale ou laver ses vitres.

Examen clinique :
Les tests d’extension-rotation sur la gauche et la droite reproduisent la douleur évoquée de façon locale en région basse cervicale. La mobilité limitée en flexion (45°), extension (20°), rotation (40°) et inflexion (5°), reproduisant la douleur. L’évaluation neurologique ne met pas en évidence de souffrance radiculaire ni de déficit sensitif ou moteur. La palpation manuelle musculaire des muscles cervicaux est douloureuse et la palpation segmentaire (manuelle statique et dynamique des articulations) du rachis cervical bas reproduit la douleur. Le reste de l’examen clinique est normal.

CONCERNANT LE DIAGNOSTIC DE CE CAS :

*

Cochez dans cette liste le ou les drapeau(x) rouge(s) identifié(s) dans ce cas clinique : (Les drapeaux rouges correspondent à des symptômes ou des signes cliniques suggérant une pathologie grave à l’origine de la douleur cervicale) :

- [ ] Signe(s) clinique(s) évoquant une une fracture traumatique ;
- [ ] Signe(s) clinique(s) évoquant une fracture ostéoporotique ;
- [ ] Signe(s) clinique(s) évoquant un cancer ;
- [ ] Signe(s) clinique(s) évoquant une infection vertébrale ;
- [ ] Signe(s) clinique(s) évoquant une myélopathie ou des déficits neurologiques sévères ou progressifs ;
- [ ] Signe(s) clinique(s) évoquant une dissection de l’artère carotide/vertébrale ;
- [ ] Signe(s) clinique(s) évoquant une hémorragie cérébrale ou une lésion intracrânienne ;
- [ ] Signe(s) clinique(s) évoquant une arthrite inflammatoire.
- [ ] Aucun signe clinique évoquant une pathologie grave

*

Cochez dans cette liste le ou les drapeau(x) jaune(s) identifié(s) dans ce cas clinique : (Les drapeaux jaunes correspondent aux facteurs de mauvais pronostic qui pourraient retarder la récupération et le rétablissement du patient)

- ☐ Un âge avancé
- ☐ Des antécédents de douleurs cervicales
- ☐ Des niveaux élevés de douleur initiale
- ☐ Des niveaux élevés d’incapacité initiale
- ☐ Des facteurs psychologiques post-traumatiques : pessimisme concernant le rétablissement ; Symptômes de stress aigu (≤ 4 semaines) ; symptômes de stress post-traumatique (≤ 4 semaines) ; humeur dépressive ou sensation de déprime due à la douleur ; anxiété ou peur de la douleur ; niveau élevé de frustration ou de colère vis-à-vis de la douleur ; passivité face à la douleur ; kinésiophobie ; activités évitées à cause de la peur de la douleur ; symptômes de stress aigu (≤ 4 semaines) ; symptômes de stress post-traumatique (≤ 4 semaines) ; humeur dépressive ou sensation de déprime due à la douleur ; anxiété ou peur de la douleur ; niveau élevé de frustration ou de colère vis-à-vis de la douleur ; passivité face à la douleur ; kinésiophobie ; activités évitées à cause de la peur de la douleur
- ☐ Aucun signe clinique évoquant un drapeau jaune

* L’anamnèse et l’examen clinique de ce cas clinique mettent-il en évidence une souffrance radiculaire ?

- ○ OUI
- ○ NON

* L’anamnèse et l’examen clinique de ce cas clinique mettent-il en évidence un déficit neurologique ?

- ○ OUI
- ○ NON

* Quel est votre diagnostic ? (une seule réponse possible)

- ○ Une cervicalgie de grade I
- ○ Une cervicalgie de grade II
- ○ Une cervicalgie de grade III
- ○ Une cervicalgie de grade IV
- ○ Autre (veuillez préciser)

____

* Est-ce que vous prenez en charge cette patiente pour ses douleurs cervicales ? (Dans le cadre de cette enquête une prise en charge sous entend l'utilisations d'alternatives thérapeutiques telles que les manipulations ou mobilisations articulaires)

- ○ OUI
- ○ NON

CONCERNANT LA PRISE EN CHARGE DE CE CAS :

* Les options thérapeutiques que je peux proposer au patient sont :

- [ ] Education du patient (continuer à mobiliser activement le rachis cervical)
- [ ] Rassurer le patient
- [ ] Une des options suivantes : 1) Prise en charge multimodale incluant des techniques de manipulations ou de mobilisations chiropratiques complétées par des exercices d’amplitude du mouvement non supervisés ; 2) Exercices d'amplitude de mouvements non supervisés.
- [ ] Une éducation du patient seule verbale ou écrite
- [ ] Un massage relaxant ou une thérapie « strain-counterstrain » (Thérapie strain-counterstrain : aussi appelée thérapie des tissus mous, qui implique une pression exercée sur un muscle du cou positionné de façon à fournir un léger étirement à ce muscle)
- [ ] Un collier cervical
- [ ] Aucune de ces options thérapeutiques

*

Réévaluation en fin de prise en charge :

Vous avez avec vu la patiente 2 fois en consultation en 10 jours. Lorsque vous lui posez la question « Comment percevez-vous la récupération de vos troubles ? » elle vous répond « Complètement améliorée ». La patiente est-elle rétablie ?

○ OUI

○ NON

* Que faites-vous :

○ J’estime que la prise en charge de ce symptôme est terminée

○ Je réfère la patiente chez son médecin

○ Je continue la prise en charge proposée

Si vous estimez que la patiente n'est pas rétablie, pour vous :

○ Le cas clinique décrit est un cas persistant (rétablissement incomplet : entamer protocole des cas persistants)

○ La gravité du cas augmente (signes de progression vers le Grade 3 : se référer aux recommandations sur les soins pour cervicalgies grade 3)

○ Le cas évolue vers une pathologie sévère (nouveaux symptômes physique, mental ou psychologique) : je dois référer la patiente à un médecin

VIGNETTES CLINIQUES - CAS CLINIQUE B

Dans cette partie, nous allons vous proposez des situations cliniques sous forme de cas clinique concernant le diagnostic et la prise en charge des douleurs cervicales en chiropraxie.

Anamnèse :
Patiente de 47 ans qui se présente avec des douleurs cervicales apparues sans traumatisme, présentent depuis environ 6 mois, car elle tricote régulièrement durant plusieurs heures. Elle cote sa douleur à 6/10. La douleur est intense au niveau de la base du cou et irradie en s'arrêtant au moignon de l'épaule droite. Elle dort mal ne trouvant pas de position de sommeil à cause de sa douleur. Elle a remarqué que prendre une douche chaude soulage ses douleurs. Elle continue son travail comme secrétaire médicale, et n'exprime pas d'incapacité particulière dans la réalisation de ses tâches quotidiennes. Elle est habituée à ressentir ce type de douleur environ 1 fois par an depuis 10 ans. Elle vous consulte en première intention sans être passée chez son médecin généraliste.

Examen clinique :
Le test d'extension-rotation sur la droite reproduit localement la douleur de la patiente en région basse cervicale. Les amplitudes de mouvement sont limitées en rotation sur la droite (30°) et inflexion droite (5°), reproduisant localement la douleur de la patiente, les autres mouvements cervicaux sont normaux. L'évaluation neurologique ne met pas en évidence de souffrance radiculaire ni de déficit sensitif ou moteur. La palpation manuelle statique et dynamique des articulations est douloureuse au niveau du rachis cervical bas. La palpation manuelle musculaire met en évidence des triggers points (points gâchettes) des muscles cervicaux à droite. Le reste de l'examen clinique est normal.

CONCERNANT LE DIAGNOSTIC DE CE CAS :

* Cochez dans cette liste le ou les drapeau(x) rouge(s) identifié(s) dans ce cas clinique : (Les drapeaux rouges correspondent à des symptômes ou des signes cliniques suggérant une pathologie grave à l'origine de la douleur cervicale) :

- ☐ Signe(s) clinique(s) évoquant une une fracture traumatique ;
- ☐ Signe(s) clinique(s) évoquant une fracture ostéoporotique ;
- ☐ Signe(s) clinique(s) évoquant un cancer ;
- ☐ Signe(s) clinique(s) évoquant une infection vertébrale ;
- ☐ Signe(s) clinique(s) évoquant une myélopathie ou des déficits neurologiques sévères ou progressifs ;
- ☐ Signe(s) clinique(s) évoquant une dissection de l'artère carotide/vertébrale ;
- ☐ Signe(s) clinique(s) évoquant une hémorragie cérébrale ou une lésion intracrânienne ;
- ☐ Signe(s) clinique(s) évoquant une arthrite inflammatoire.
- ☐ Aucun signe clinique évoquant une pathologie grave

* Cochez dans cette liste le ou les drapeau(x) jaune(s) identifié(s) dans ce cas clinique : (Les drapeaux jaunes correspondent aux facteurs de mauvais pronostic qui pourraient retarder la récupération et le rétablissement du patient.

- [ ] Un âge avancé
- [ ] Des antécédents de douleurs cervicales
- [ ] Des niveaux élevés de douleur initiale
- [ ] Des niveaux élevés d'incapacité initiale
- [ ] Des facteurs psychologiques post-traumatiques : pessimisme concernant le rétablissement ; Symptômes de stress aigu (≤ 4 semaines) ; symptômes de stress post-traumatique (≤ 4 semaines) ; humeur dépressive ou sensation de déprime due à la douleur ; anxiété ou peur de la douleur ; niveau élevé de frustration ou de colère vis-à-vis de la douleur ; passivité face à la douleur ; kinésiophobie ; activités évitées à cause de la peur de la douleur ; symptômes de stress aigu (≤ 4 semaines) ; symptômes de stress post-traumatique (≤ 4 semaines) ; humeur dépressive ou sensation de déprime due à la douleur ; anxiété ou peur de la douleur ; niveau élevé de frustration ou de colère vis-à-vis de la douleur ; passivité face à la douleur ; kinésiophobie ; activités évitées à cause de la peur de la douleur
- [ ] Aucun signe clinique évoquant un drapeau jaune

* L'anamnèse et l'examen clinique de ce cas clinique mettent-il en évidence une souffrance radiculaire ?

- ○ OUI
- ○ NON

* L'anamnèse et l'examen clinique de ce cas clinique mettent-il en évidence un déficit neurologique ?

- ○ OUI
- ○ NON

* Quel est votre diagnostic ? (une seule réponse possible)

- ○ Une cervicalgie de grade I
- ○ Une cervicalgie de grade II
- ○ Une cervicalgie de grade III
- ○ Une cervicalgie de grade IV
- ○ Autre (veuillez préciser)

____

* Est-ce que vous prenez en charge cette patiente pour ses douleurs cervicales ? (Dans le cadre de cette enquête une prise en charge sous entend l'utilisations d'alternatives thérapeutiques telles que les manipulations ou mobilisations articulaires)

- ○ OUI
- ○ NON

CONCERNANT LA PRISE EN CHARGE DE CE CAS :

* Les options thérapeutiques que je peux proposer au patient sont :

- [ ] Education du patient (continuer à mobiliser activement le rachis cervical)
- [ ] Rassurer le patient
- [ ] Une des options suivantes : 1) Prise en charge multimodale incluant des techniques de manipulations ou de mobilisations chiropratiques complétées par des exercices d'amplitude du mouvement non supervisés ; 2) Exercices d'amplitude de mouvements non supervisés; 3) Activités physiques douces complémentaires (ex: qijong ou iyengar yoga)
- [ ] Une éducation du patient seule verbale ou écrite
- [ ] Un massage relaxant ou une thérapie « strain-counterstrain » (Thérapie strain-counterstrain : aussi appelée thérapie des tissus mous, qui implique une pression exercée sur un muscle du cou positionné de façon à fournir un léger étirement à ce muscle)
- [ ] Un collier cervical
- [ ] Aucune de ces options thérapeutiques

* *Réévaluation en fin de prise en charge :*

Vous avez avec vu la patiente 3 fois en consultation en l'espace d'un mois. Lorsque vous lui posez la question « Comment percevez-vous la récupération de vos troubles ? » elle vous répond « Améliorée ». La patiente est-elle rétablie ?

- ○ OUI
- ○ NON

* Que faites-vous :

- ○ J'estime que la prise en charge de ce symptôme est terminée
- ○ Je réfère la patiente chez son médecin
- ○ Je continue la prise en charge proposée

* Si non, pour quelle(s) raison(s) :

- [ ] Le cas clinique décrit est un cas persistant (rétablissement incomplet : entamer protocole des cas persistants)
- [ ] La gravité du cas augmente (signes de progression vers le Grade 3 : se référer aux recommandations sur les soins pour cervicalgies grade 3)
- [ ] Le cas évolue vers une pathologie sévère (nouveaux symptômes physique, mental ou psychologique) : je dois référer la patiente à un médecin

Enquête nationale stagiaires en centres de soins (PILOTE) - Diagnostic & prise en charge des cervicalgies en chiropraxie

VIGNETTES CLINIQUES - CAS CLINIQUE B (suite)

Dans cette partie, nous allons vous proposez des situations cliniques sous forme de cas clinique

concernant le diagnostic et la prise en charge des douleurs cervicales en chiropraxie.

Anamnèse :
Deux ans après, cette même patiente de 49 ans revient vous voir avec un nouvel épisode de douleur cervicale associée à une irradiation au niveau de la face latérale du bras et de l'avant-bras après avoir peint un plafond la veille (dans les 24h précédentes), positionnée sur une échelle la tête en extension. La douleur se situe au niveau de l'arrière du cou et descend dans son bras droit. Elle a très mal dormi la nuit passée à cause de sa douleur. Elle cote sa douleur à 7/10, et décrit ses douleurs dans le bras comme des décharges électriques. En revanche, elle ne ressent aucune perte de force, aucune perte de sensibilité dans son bras, ni de symptôme particulier dans les jambes. Lorsqu'elle se présente à vous, elle adapte une position antalgique la tête légèrement penchée sur la gauche, mais elle ne ressent pas la nécessité de se tenir le cou avec ses mains. Elle a pris un anti-inflammatoire non stéroïdien, 2h avant de venir vous voir, et cela l'a soulagée en diminuant sa douleur à 5/10. Elle vous explique avoir eu des difficultés pour lire son journal ce matin, et un accompagnateur a dû la conduire jusqu'à vous, ne pouvant conduire. Elle ne relate aucun autre traumatisme pouvant expliquer sa symptomatologie. Jusqu'alors, elle n'a jamais ressenti d'irradiation dans le bras et son dernier épisode de cervicalgie remonte à un an. Ce nouveau symptôme l'inquiète en la rendant pessimiste concernant sa récupération. Elle vous consulte en première intention sans être passée chez son médecin généraliste.

Examen clinique :
Toutes les amplitudes de mouvements cervicaux sont douloureuses et limitées (inflexion 5°, rotation 25°, flexion 50°, extension 5°). Le test d'extension-rotation cervicale sur la droite reproduit l'irradiation dans le membre supérieur droit. Les tests de traction (ULLT) des nerf radial et médian sont positifs à droite. Vous mettez en évidence un déficit neurologique de la racine nerveuse C6 droite avec un myotome C6 (flexion bras, extension poignet, flexion des doigts) évalué à 4/5 sur l'échelle MRC (échelle d'évaluation manuelle de la force musculaire), un réflexe ostéotendineux C6 évalué à 1+ sur l'échelle Wexler (échelle d'évaluation des réflexes ostéotendineux) à droite, une hypoesthésie du territoire C6 à droite. Pas de déficit neurologique mis en évidence lors de l'évaluation du membre supérieur controlatéral. La palpation manuelle des muscles cervicaux est douloureuse et la palpation segmentaire dynamique et statique du rachis cervical bas reproduit la douleur. L'examen neurologique central est négatif tout comme le test de Romberg et les réflexes cutanées plantaires. Le reste de l'examen clinique est normal.

CONCERNANT LE DIAGNOSTIC DE CE CAS :

* Cochez dans cette liste le ou les drapeau(x) rouge(s) identifié(s) dans ce cas clinique : (Les drapeaux rouges correspondent à des symptômes ou des signes cliniques suggérant une pathologie grave à l'origine de la douleur cervicale) :

- ☐ Signe(s) clinique(s) évoquant une une fracture traumatique ;
- ☐ Signe(s) clinique(s) évoquant une fracture ostéoporotique ;
- ☐ Signe(s) clinique(s) évoquant un cancer ;
- ☐ Signe(s) clinique(s) évoquant une infection vertébrale ;
- ☐ Signe(s) clinique(s) évoquant une myélopathie ou des déficits neurologiques sévères ou progressifs ;
- ☐ Signe(s) clinique(s) évoquant une dissection de l'artère carotide/vertébrale ;
- ☐ Signe(s) clinique(s) évoquant une hémorragie cérébrale ou une lésion intracrânienne ;
- ☐ Signe(s) clinique(s) évoquant une arthrite inflammatoire.
- ☐ Aucun signe clinique évoquant une pathologie grave

* Cochez dans cette liste le ou les drapeau(x) jaune(s) identifié(s) dans ce cas clinique : (Les drapeaux jaunes correspondent aux facteurs de mauvais pronostic qui pourraient retarder la récupération et le rétablissement du patient.

- ☐ Un âge avancé
- ☐ Des antécédents de douleurs cervicales
- ☐ Des niveaux élevés de douleur initiale
- ☐ Des niveaux élevés d’incapacité initiale
- ☐ Des facteurs psychologiques post-traumatiques : pessimisme concernant le rétablissement ; Symptômes de stress aigu (≤ 4 semaines) ; symptômes de stress post-traumatique (≤ 4 semaines) ; humeur dépressive ou sensation de déprime due à la douleur ; anxiété ou peur de la douleur ; niveau élevé de frustration ou de colère vis-à-vis de la douleur ; passivité face à la douleur ; kinésiophobie ; activités évitées à cause de la peur de la douleur ; symptômes de stress aigu (≤ 4 semaines) ; symptômes de stress post-traumatique (≤ 4 semaines) ; humeur dépressive ou sensation de déprime due à la douleur ; anxiété ou peur de la douleur ; niveau élevé de frustration ou de colère vis-à-vis de la douleur ; passivité face à la douleur ; kinésiophobie ; activités évitées à cause de la peur de la douleur
- ☐ Aucun signe clinique évoquant un drapeau jaune

* L’anamnèse et l’examen clinique de ce cas clinique mettent-il en évidence une souffrance radiculaire ?

- ○ OUI
- ○ NON

* L’anamnèse et l’examen clinique de ce cas clinique mettent-il en évidence un déficit neurologique ?

- ○ OUI
- ○ NON

* Quel est votre diagnostic ? (une seule réponse possible)

- ○ Une cervicalgie de grade I
- ○ Une cervicalgie de grade II
- ○ Une cervicalgie de grade III
- ○ Une cervicalgie de grade IV
- ○ Autre (veuillez préciser)

* A ce stade de la consultation chiropratique, les options thérapeutiques que je peux proposer au patient sont :

- ☐ Education du patient (continuer à mobiliser activement le rachis cervical)
- ☐ Rassurer le patient
- ☐ Exercices d'étirements, de renforcement, de stabilisation, de mobilité, de relaxation du cou gradés et supervisés
- ☐ Référer le patient pour un avis médical
- ☐ Manipulations ou mobilisations adaptées à la condition physique du patient
- ☐ Une éducation du patient seule verbale ou écrite
- ☐ Une traction intermittente
- ☐ Un Collier cervical
- ☐ Aucune de ces options thérapeutiques

CONCERNANT LA PRISE EN CHARGE DE CE CAS :

* Les options thérapeutiques que je peux proposer au patient en l'absence de contre indication médicale sont :

- ☐ Education du patient (continuer à mobiliser activement le rachis cervical)
- ☐ Rassurer le patient
- ☐ Exercices d'étirements, de renforcement, de stabilisation, de mobilité, de relaxation du cou gradés et supervisés
- ☐ Manipulations ou mobilisations adaptées à la condition physique du patient
- ☐ Une éducation du patient seule verbale ou écrite
- ☐ Une traction intermittente
- ☐ Un Collier cervical
- ☐ Aucune de ces options thérapeutiques

* *Réévaluation en fin de prise en charge :*

Vous avez avec vu la patiente 4 fois en consultation en l'espace de 1 mois et demi. Lorsque vous lui posez la question « Comment percevez-vous la récupération de vos troubles ? » elle vous répond « Améliorée ». La patiente est-elle rétablie ?

- ○ OUI
- ○ NON

* Que faites-vous :

- ○ J'estime que la prise en charge de ce symptôme est terminée
- ○ Je réfère la patiente chez son médecin
- ○ Je continue la prise en charge proposée

* Si non, pour quelle(s) raison(s) :

- Le cas clinique décrit est un cas persistant (rétablissement incomplet : entamer protocole des cas persistants)
- La gravité du cas augmente (signes de progression vers le Grade 3 : se référer aux recommandations sur les soins pour cervicalgies grade 3)
- Le cas évolue vers une pathologie sévère (nouveaux symptômes physique, mental ou psychologique) : je dois référer la patiente à un médecin

Enquête nationale stagiaires en centres de soins (PILOTE) - Diagnostic & prise en charge des cervicalgies en chiropraxie

VIGNETTES CLINIQUES - CAS CLINIQUE C

Dans cette partie, nous allons vous proposez des situations cliniques sous forme de cas clinique concernant le diagnostic et la prise en charge des douleurs cervicales en chiropraxie.

Anamnèse :
Patient de 52 ans qui consulte pour des douleurs cervicales à la suite d'un accident de la voie publique par collision arrière à environ 110 km/h. Lors de l'accident, le patient était légèrement désorienté, il n'a pas perdu connaissance. Les pompiers l'ont transporté allongé avec un collier de maintien cervical rigide jusqu'aux urgences.
Une prise en charge aux urgences et des radiographies ont été effectuées directement après l'accident mettant en évidence une fracture cervicale non déplacée au niveau du processus épineux de C5. Les urgences n'ont pas gardé le patient en surveillance. On lui a conseillé de conserver le collier cervical, et de rentrer accompagné chez lui. Il a eu très mal aux cervicales après l'accident et cotait ses douleurs à 9/10. Depuis l'accident, le patient garde une amplitude de mouvement limitée en rotation (5°) et inflexion (10°) sur la droite, en extension (5°). Le reste des amplitudes de mouvement est conservé.
Quinze jours après cet accident, il vous consulte car il a très peur que ses limitations de mouvement et sa douleur persistent. Il est très inquiet concernant l'évolution de ses douleurs. Ses douleurs l'empêchent de vivre normalement, il a arrêté toute activité physique et ne peut pas conduire ce qui l'embête beaucoup.
Il cote ses douleurs à 5/10 en fond douloureux étant sous AINS et 8/10 en pic aigu de douleur quand il tourne la tête à droite, ou qu'il se trouve dans la position tête penchée en avant. Il décrit la douleur comme étant localisée au niveau de la base du cou et quand il regarde trop vite sur le côté, il ressent de l'électricité au niveau de son avant-bras et du pouce à droite. Il n'évoque pas de symptôme particulier dans les jambes. Le repos et les anti-inflammatoires non stéroïdiens le soulagent.

Examen clinique :
Les amplitudes de mouvement en rotation sur la droite (5°) et en extension cervicale (5°) sont limitées et reproduisent la douleur et les irradiations. L'évaluation des myotomes, dermatomes et réflexes ne mettent pas en évidence un déficit sensitif ou moteur. Les tests de traction (ULLT) des nerfs radial et médian sont positifs à droite. La douleur est impulsive. Celle des muscles cervicaux est douloureuse et la palpation segmentaire statique du rachis cervical bas reproduit la douleur. La palpation manuelle des processus épineux cervicaux (palpation médiane du rachis) se révèle douloureuse en C5. L'examen neurologique central est négatif tout comme le test de Romberg et les réflexes cutanés plantaires. Le reste de l'examen clinique est normal.

CONCERNANT LE DIAGNOSTIC DE CE CAS :

* Cochez dans cette liste le ou les drapeau(x) rouge(s) identifié(s) dans ce cas clinique : (Les drapeaux rouges correspondent à des symptômes ou des signes cliniques suggérant une pathologie grave à l'origine de la douleur cervicale) :

- [ ] Signe(s) clinique(s) évoquant une une fracture traumatique ;
- [ ] Signe(s) clinique(s) évoquant une fracture ostéoporotique ;
- [ ] Signe(s) clinique(s) évoquant un cancer ;
- [ ] Signe(s) clinique(s) évoquant une infection vertébrale ;
- [ ] Signe(s) clinique(s) évoquant une myélopathie ou des déficits neurologiques sévères ou progressifs ;
- [ ] Signe(s) clinique(s) évoquant une dissection de l'artère carotide/vertébrale ;
- [ ] Signe(s) clinique(s) évoquant une hémorragie cérébrale ou une lésion intracrânienne ;
- [ ] Signe(s) clinique(s) évoquant une arthrite inflammatoire.
- [ ] Aucun signe clinique évoquant une pathologie grave

* Cochez dans cette liste le ou les drapeau(x) jaune(s) identifié(s) dans ce cas clinique : (Les drapeaux jaunes correspondent aux facteurs de mauvais pronostic qui pourraient retarder la récupération et le rétablissement du patient.

- [ ] Un âge avancé
- [ ] Des antécédents de douleurs cervicales
- [ ] Des niveaux élevés de douleur initiale
- [ ] Des niveaux élevés d'incapacité initiale
- [ ] Des facteurs psychologiques post-traumatiques : pessimisme concernant le rétablissement ; Symptômes de stress aigu (≤ 4 semaines) ; symptômes de stress post-traumatique (≤ 4 semaines) ; humeur dépressive ou sensation de déprime due à la douleur ; anxiété ou peur de la douleur ; niveau élevé de frustration ou de colère vis-à-vis de la douleur ; passivité face à la douleur ; kinésiophobie ; activités évitées à cause de la peur de la douleur ; symptômes de stress aigu (≤ 4 semaines) ; symptômes de stress post-traumatique (≤ 4 semaines) ; humeur dépressive ou sensation de déprime due à la douleur ; anxiété ou peur de la douleur ; niveau élevé de frustration ou de colère vis-à-vis de la douleur ; passivité face à la douleur ; kinésiophobie ; activités évitées à cause de la peur de la douleur
- [ ] Aucun signe clinique évoquant un drapeau jaune

* L'anamnèse et l'examen clinique de ce cas clinique mettent-il en évidence une souffrance radiculaire ?

- ○ OUI
- ○ NON

* L'anamnèse et l'examen clinique de ce cas clinique mettent-il en évidence un déficit neurologique ?

- ○ OUI
- ○ NON

* Quel est votre diagnostic ? (une seule réponse possible)

- ◯ Une cervicalgie de grade I
- ◯ Une cervicalgie de grade II
- ◯ Une cervicalgie de grade III
- ◯ Une cervicalgie de grade IV
- ◯ Autre (veuillez préciser)

* Est-ce que vous prenez en charge cette patiente pour ses douleurs cervicales ? (Dans le cadre de cette enquête une prise en charge sous entend l'utilisations d'alternatives thérapeutiques telles que les manipulations ou mobilisations articulaires)

- ◯ OUI
- ◯ NON

Enquête nationale stagiaires en centres de soins (PILOTE) - Diagnostic & prise en charge des cervicalgies en chiropraxie

DETERMINANTS D'UTILISATIONS DES RECOMMANDATIONS DE BONNE PRATIQUES (1/7)

Dans votre pratique quotidienne, vous vous décririez comme :

* Soutenu(e) par les autres professionnels de la santé :

- ◯ D'accord
- ◯ Plutôt d'accord
- ◯ Plutôt pas d'accord
- ◯ Pas d'accord

* Soutenu(e) par les politiques de santé :

- ◯ D'accord
- ◯ Plutôt d'accord
- ◯ Plutôt pas d'accord
- ◯ Pas d'accord

* Sous pression au centre de soins (IFEC) :

- D'accord
- Plutôt d'accord
- Plutôt pas d'accord
- Pas d'accord

* En compétition avec les autres professionnels de la santé :

- D'accord
- Plutôt d'accord
- Plutôt pas d'accord
- Pas d'accord

* Intéressé(e) par les données scientifiques publiées :

- D'accord
- Plutôt d'accord
- Plutôt pas d'accord
- Pas d'accord

* Inférieur(e) aux autres professionnels de la santé :

- D'accord
- Plutôt d'accord
- Plutôt pas d'accord
- Pas d'accord

* Etant à la recherche d’outils et techniques récents :

- D'accord
- Plutôt d'accord
- Plutôt pas d'accord
- Pas d'accord

* Frustré(e), angoissé(e), démuni(e) pour aider mon patient :

- D'accord
- Plutôt d'accord
- Plutôt pas d'accord
- Pas d'accord

* Prenant en charge beaucoup de patients cervicalgiques :

- D'accord
- Plutôt d'accord
- Plutôt pas d'accord
- Pas d'accord

* Ayant une vision critique du système de soin médical :

- D'accord
- Plutôt d'accord
- Plutôt pas d'accord
- Pas d'accord

* Etant attentif au diagnostic des facteurs biopsychosociaux :

- D'accord
- Plutôt d'accord
- Plutôt pas d'accord
- Pas d'accord

* Ayant peur de manquer un détail important du diagnostic ou du pronostic du patient :

- D'accord
- Plutôt d'accord
- Plutôt pas d'accord
- Pas d'accord

* Non adhérent aux prises en charge basées sur des preuves scientifiques (evidence-based medecine) :

- D'accord
- Plutôt d'accord
- Plutôt pas d'accord
- Pas d'accord

* Un utilisateur des recommandations de bonnes pratiques :

- D'accord
- Plutôt d'accord
- Plutôt pas d'accord
- Pas d'accord

* Ayant peur de la douleur :

- D'accord
- Plutôt d'accord
- Plutôt pas d'accord
- Pas d'accord

* Ayant peur de rendre mes patients insatisfaits :

- D'accord
- Plutôt d'accord
- Plutôt pas d'accord
- Pas d'accord

* Alerte sur le diagnostic et la prise en charge des drapeaux jaunes (facteurs de passage à la chronicité) :

- D'accord
- Plutôt d'accord
- Plutôt pas d'accord
- Pas d'accord

Enquête nationale stagiaires en centres de soins (PILOTE) - Diagnostic & prise en charge des cervicalgies en chiropraxie

DETERMINANTS D'UTILISATIONS DES RECOMMANDATIONS DE BONNE PRATIQUES (2/7)

Que pensez-vous de la prise en charge pluridisciplinaire des troubles musculosquelettiques ?

* J’ai une expérience positive d’une précédente prise en charge pluridisciplinaire :

- D'accord
- Plutôt d'accord
- Plutôt pas d'accord
- Pas d'accord

* Je ressens un manque de soutien de la part des autres professionnels de la santé :

- D'accord
- Plutôt d'accord
- Plutôt pas d'accord
- Pas d'accord

* J’ai une expérience négative d’une précédente prise en charge pluridisciplinaire :

- D'accord
- Plutôt d'accord
- Plutôt pas d'accord
- Pas d'accord

* Mon approche clinique ou mon organisation sont différentes des autres collaborateurs, par conséquent je ne peux pas faire de prise en charge pluridisciplinaire :

- D'accord
- Plutôt d'accord
- Plutôt pas d'accord
- Pas d'accord

* Je serais plus ouvert(e) à la prise en charge pluridisciplinaire si j’étais rémunéré(e) pour cela :

- D'accord
- Plutôt d'accord
- Plutôt pas d'accord
- Pas d'accord

* Je ne suis pas certain du rôle des autres professionnels de la santé, par conséquent je ne pratique pas la pluridisciplinarité :

- D'accord
- Plutôt d'accord
- Plutôt pas d'accord
- Pas d'accord

* Je préfère prendre mes propres décisions sans l’aide de confrères ou de recommandations de bonnes pratiques :

- D'accord
- Plutôt d'accord
- Plutôt pas d'accord
- Pas d'accord

DETERMINANTS D'UTILISATIONS DES RECOMMANDATIONS DE BONNE PRATIQUES (3/7)

Avec quel type de patients utilisez-vous les recommandations de bonne pratique ?

* Tous les patients sans exception :

- D'accord
- Plutôt d'accord
- Plutôt pas d'accord
- Pas d'accord

* Aucun patient :

- D'accord
- Plutôt d'accord
- Plutôt pas d'accord
- Pas d'accord

* Un cas clinique complexe :

- D'accord
- Plutôt d'accord
- Plutôt pas d'accord
- Pas d'accord

* Un patient avec des comorbidités (par ex : exposition à d’autres maladies : maladie cardio-vasculaire, diabète, cancer, …) :

- D'accord
- Plutôt d'accord
- Plutôt pas d'accord
- Pas d'accord

* Un patient de longue date :

- D'accord
- Plutôt d'accord
- Plutôt pas d'accord
- Pas d'accord

* Un patient avec des symptômes ou signes cliniques suggérant une pathologie grave (présence de drapeaux rouges) :

- D'accord
- Plutôt d'accord
- Plutôt pas d'accord
- Pas d'accord

* Un patient vivant dans une situation socio-économique difficile :

- D'accord
- Plutôt d'accord
- Plutôt pas d'accord
- Pas d'accord

* Un patient anxieux pour le rassurer :

- D'accord
- Plutôt d'accord
- Plutôt pas d'accord
- Pas d'accord

Enquête nationale stagiaires en centres de soins (PILOTE) - Diagnostic & prise en charge des cervicalgies en chiropraxie

DETERMINANTS D'UTILISATIONS DES RECOMMANDATIONS DE BONNE PRATIQUES (4/7)

D'après les déterminants listés ci-dessous, dites-nous lesquels pourraient favoriser ou empêcher votre utilisation des recommandations de bonne pratique :

* Le manque d'indemnisation financière lié à leur utilisation :

- M'empêche d'utiliser les recommandations de bonnes pratique
- Favorise mon utilisation des recommandations de bonne pratique
- Ce déterminant n'influence pas mon utilisation des recommandations de bonne pratique

* L'utilisation des recommandations par des pairs :

- M'empêche d'utiliser les recommandations de bonnes pratique
- Favorise mon utilisation des recommandations de bonne pratique
- Ce déterminant n'influence pas mon utilisation des recommandations de bonne pratique

* Le modèle biopsychosocial des TMS :

- M’empêche d’utiliser les recommandations de bonnes pratique
- Favorise mon utilisation des recommandations de bonne pratique
- Ce déterminant n’influence pas mon utilisation des recommandations de bonne pratique

Enquête nationale stagiaires en centres de soins (PILOTE) - Diagnostic & prise en charge des cervicalgies en chiropraxie

DETERMINANTS D'UTILISATIONS DES RECOMMANDATIONS DE BONNE PRATIQUES (5/7)

Concernant l’utilité ou non des recommandations de bonne pratique, d’après vous les recommandations de bonnes pratiques:

* Sont source de nouvelles informations :

- D'accord
- Plutôt d'accord
- Plutôt pas d'accord
- Pas d'accord

Ne sont pas assez pratiques pour une utilisation quotidienne :

- D'accord
- Plutôt d'accord
- Plutôt pas d'accord
- Pas d'accord

* Permettent d’augmenter la qualité des soins :

- D'accord
- Plutôt d'accord
- Plutôt pas d'accord
- Pas d'accord

* Contiennent trop d’informations :

- D'accord
- Plutôt d'accord
- Plutôt pas d'accord
- Pas d'accord

* Permettent un langage standardisé avec les autres professionnels de la santé :

- D'accord
- Plutôt d'accord
- Plutôt pas d'accord
- Pas d'accord

* Sont contradictoires avec les attentes du patient :

- D'accord
- Plutôt d'accord
- Plutôt pas d'accord
- Pas d'accord

* Permettent de communiquer avec les autres professionnels de la santé :

- D'accord
- Plutôt d'accord
- Plutôt pas d'accord
- Pas d'accord

* Déshumanisent mes relations avec mon patients :

- D'accord
- Plutôt d'accord
- Plutôt pas d'accord
- Pas d'accord

* Fournissent de bonnes explications pour communiquer avec mon patient :

- D'accord
- Plutôt d'accord
- Plutôt pas d'accord
- Pas d'accord

* Ne sont pas adaptées à la philosophie chiropratique, à l’histoire de la chiropratique :

- D'accord
- Plutôt d'accord
- Plutôt pas d'accord
- Pas d'accord

* Orientent vers des traitements valides et utiles :

- D'accord
- Plutôt d'accord
- Plutôt pas d'accord
- Pas d'accord

* Donnent des conseils d'éducation adressés aux patients face à leur douleur :

- D'accord
- Plutôt d'accord
- Plutôt pas d'accord
- Pas d'accord

* Permettent de m'aider dans la prise en charge des drapeaux jaunes :

- D'accord
- Plutôt d'accord
- Plutôt pas d'accord
- Pas d'accord

* Permettent de rassurer mes patients anxieux :

- D'accord
- Plutôt d'accord
- Plutôt pas d'accord
- Pas d'accord

* Limitent ma communication avec mes collègues ou mes patients :

- D'accord
- Plutôt d'accord
- Plutôt pas d'accord
- Pas d'accord

* Permettent de donner des objectifs à mes patients :

- D'accord
- Plutôt d'accord
- Plutôt pas d'accord
- Pas d'accord

* Permettent de limiter le coût des soins :

- D'accord
- Plutôt d'accord
- Plutôt pas d'accord
- Pas d'accord

* Permettent de limiter les risques liés aux soins :

- D'accord
- Plutôt d'accord
- Plutôt pas d'accord
- Pas d'accord

* Sont une source de réponses aux questions des patients :

- D'accord
- Plutôt d'accord
- Plutôt pas d'accord
- Pas d'accord

* Proposent un traitement idéal non adapté à mes patients :

- D'accord
- Plutôt d'accord
- Plutôt pas d'accord
- Pas d'accord

* Ont des conséquences sur mes relations avec mes patients (perte de patientèle, insatisfaction) :

- D'accord
- Plutôt d'accord
- Plutôt pas d'accord
- Pas d'accord

DETERMINANTS D'UTILISATIONS DES RECOMMANDATIONS DE BONNE PRATIQUES (6/7)

Pour moi, les recommandations de bonnes pratiques sont :

* Trop théoriques :

- D'accord
- Plutôt d'accord
- Plutôt pas d'accord
- Pas d'accord

* Compréhensibles :

- D'accord
- Plutôt d'accord
- Plutôt pas d'accord
- Pas d'accord

* Trop longues, fastidieuses :

- D'accord
- Plutôt d'accord
- Plutôt pas d'accord
- Pas d'accord

* Manquent de niveau de preuve :

- D'accord
- Plutôt d'accord
- Plutôt pas d'accord
- Pas d'accord

* Trop standardisées, génériques, pas applicables à tous les patients :

- D'accord
- Plutôt d'accord
- Plutôt pas d'accord
- Pas d'accord

* Evaluées par des pairs :

- D'accord
- Plutôt d'accord
- Plutôt pas d'accord
- Pas d'accord

* Incompatibles avec mon approche clinique :

- D'accord
- Plutôt d'accord
- Plutôt pas d'accord
- Pas d'accord

* Interprétables de différentes façons :

- D'accord
- Plutôt d'accord
- Plutôt pas d'accord
- Pas d'accord

* Trop nombreuses :

- D'accord
- Plutôt d'accord
- Plutôt pas d'accord
- Pas d'accord

* Dépassées et manquant de données scientifiques récentes :

- D'accord
- Plutôt d'accord
- Plutôt pas d'accord
- Pas d'accord

* Pertinentes :

- D'accord
- Plutôt d'accord
- Plutôt pas d'accord
- Pas d'accord

* Claires, concises :

- D'accord
- Plutôt d'accord
- Plutôt pas d'accord
- Pas d'accord

* Manquent de conseils pratiques :

- D'accord
- Plutôt d'accord
- Plutôt pas d'accord
- Pas d'accord

* Accessibles :

- D'accord
- Plutôt d'accord
- Plutôt pas d'accord
- Pas d'accord

* Un gain de temps :

- D'accord
- Plutôt d'accord
- Plutôt pas d'accord
- Pas d'accord

* Manquent de cohérence dans leurs méthodologies :

- D'accord
- Plutôt d'accord
- Plutôt pas d'accord
- Pas d'accord

* Utiles :

- D'accord
- Plutôt d'accord
- Plutôt pas d'accord
- Pas d'accord

* Restrictives :

- D'accord
- Plutôt d'accord
- Plutôt pas d'accord
- Pas d'accord

* Adaptées à ma pratique quotidienne :

- D'accord
- Plutôt d'accord
- Plutôt pas d'accord
- Pas d'accord

* Une perte d’autonomie :

- D'accord
- Plutôt d'accord
- Plutôt pas d'accord
- Pas d'accord

* Obligatoires :

- D'accord
- Plutôt d'accord
- Plutôt pas d'accord
- Pas d'accord

Enquête nationale stagiaires en centres de soins (PILOTE) - Diagnostic & prise en charge des cervicalgies en chiropraxie

DETERMINANTS D'UTILISATIONS DES RECOMMANDATIONS DE BONNE PRATIQUES (7/7)

Pour utiliser les recommandations de bonnes pratiques, je manque de connaissances ou d’entrainement sur :

* Leur contenu :

- D'accord
- Plutôt d'accord
- Plutôt pas d'accord
- Pas d'accord

* Les tests diagnostiques qu’elles proposent :

- D'accord
- Plutôt d'accord
- Plutôt pas d'accord
- Pas d'accord

* La prise en charge de facteurs de passage à la chronicité (drapeaux jaunes) :

- D'accord
- Plutôt d'accord
- Plutôt pas d'accord
- Pas d'accord

* La terminologie utilisée (notamment le terme de « non spécifique ») :

- D'accord
- Plutôt d'accord
- Plutôt pas d'accord
- Pas d'accord

* Les traitements qu’elles proposent :

- D'accord
- Plutôt d'accord
- Plutôt pas d'accord
- Pas d'accord

* La physiopathologie des troubles musculosquelettiques :

- D'accord
- Plutôt d'accord
- Plutôt pas d'accord
- Pas d'accord

Enquête nationale stagiaires en centres de soins (PILOTE) - Diagnostic & prise en charge des cervicalgies en chiropraxie

QUESTIONNAIRE SOCIO-DEMOGRAPHIQUE

* Vous êtes :

- Un homme
- Une femme
- Autre (veuillez préciser)

* Quel est votre âge ? (saisir le nombre correspondant à vôtre âge)

* Combien de consultations comptabilisez vous au centre de soins ?

- ◯ Entre 0 et 100 consultations
- ◯ Entre 101 et 200 consultations
- ◯ Entre 201 et 300 consultations
- ◯ Entre 301 et 360 consultations

* Dans votre pratique quotidienne, utilisez-vous les recommandations de bonne pratique publiées :

- ◯ OUI
- ◯ NON

* Vous les utilisez pour poser un diagnostic :

- ◯ TOUJOURS
- ◯ SOUVENT
- ◯ PARFOIS
- ◯ RAREMENT
- ◯ JAMAIS

* Vous les utilisez pour choisir la prise en charge proposée au patient :

- ◯ TOUJOURS
- ◯ SOUVENT
- ◯ PARFOIS
- ◯ RAREMENT
- ◯ JAMAIS

* Vous les utilisez pour évaluer le pronostic du patient :

- ◯ TOUJOURS
- ◯ SOUVENT
- ◯ PARFOIS
- ◯ RAREMENT
- ◯ JAMAIS

* Quelles recommandations utilisez vous dans votre pratique ?

- [ ] Je n'en utilise pas
- [ ] Recommandations NICE
- [ ] Recommandations Canadienne du Groupe OPTIMA
- [ ] Recommandations de l'AFC, publiées pour les chiropracteurs et labellisées par la Haute Autorité de Santé
- [ ] Recommandations canadiennes (Neck pain task force, OPTIMa, …)
- [ ] Recommandations Américaine de l'American College of Physicians
- [ ] Guide de Bonne Pratique Clinique Belge (KCE)
- [ ] Recommandations anglaise du Scottish Intercollegiate Guidelines Network (SIGN)
- [ ] Recommandations Danoises
- [ ] Recommandations de la SOFEC (SOciété Franco-Européenne de Chiropratique)
- [ ] Recommandations générales (toutes professions confondues) de la HAS (Haute Autorité de Santé)
- [ ] Autre (veuillez préciser)

* Avez-vous suivi un enseignement concernant l'utilisation de recommandations de bonne pratique ?

- ○ OUI
- ○ NON

Enquête nationale stagiaires en centres de soins (PILOTE) - Diagnostic & prise en charge des cervicalgies en chiropraxie

Merci à tous pour votre participation !

Vous êtes arrivé(e) à la fin de cette enquête et je vous en remercie grandement. Si vous souhaitez participer au tirage au sort pour gagner votre participation à un séminaire chiro offert par l'AFC envoyez votre NOM/PRENOM/ADRESSE MAIL DE CONTACT à cette adresse (tirageausort2021@ifec.net).

Et n'oubliez pas vous pouvez me contacter ici dsorondo@ifec.net pour le SAV du questionnaire ☺
